# Alteration of the gut microbiota’s composition and metabolic output correlates with COVID-19-like severity in obese NASH hamsters

**DOI:** 10.1080/19490976.2022.2100200

**Published:** 2022-07-13

**Authors:** Valentin Sencio, Nicolas Benech, Cyril Robil, Lucie Deruyter, Séverine Heumel, Arnaud Machelart, Thierry Sulpice, Antonin Lamazière, Corinne Grangette, François Briand, Harry Sokol, François Trottein

**Affiliations:** a CHU Lille, Institut Pasteur de Lille, U1019 - UMR 9017 - CIIL - Center for Infection and Immunity of Lille, Univ. Lille, CNRS, Inserm, Lille, France; b UMR 9017, Centre National de la Recherche Scientifique (CNRS), Lille, France; c Institut National de la Santé et de la Recherche Médicale (Inserm) U1019, Lille, France; d Centre Hospitalier Universitaire de Lille, Lille, France; e Institut Pasteur de Lille, Lille, France; f Inserm, Centre de Recherche Saint-Antoine, CRSA, AP-HP, Saint Antoine Hospital, Gastroenterology department, Sorbonne Université, Paris, France; g Paris Center for Microbiome Medicine, Fédération Hospitalo-Universitaire, Paris, France; h Physiogenex, Escalquens, France; i UMR1319 Micalis & AgroParisTech, Institut National de la Recherche Agronomique (INRAE), Jouy en Josas, France

**Keywords:** SARS-CoV-2, hamsters, gut microbiota, obesity, NASH, COVID-19

## Abstract

Obese patientss with nonalcoholic steatohepatitis (NASH) are particularly prone to developing severe forms of coronavirus disease 19 (COVID-19). The gut-to-lung axis is critical during viral infections of the respiratory tract, and a change in the gut microbiota’s composition might have a critical role in disease severity. Here, we investigated the consequences of infection with severe acute respiratory syndrome coronavirus 2 (SARS-CoV-2) on the gut microbiota in the context of obesity and NASH. To this end, we set up a nutritional model of obesity with dyslipidemia and NASH in the golden hamster, a relevant preclinical model of COVID-19. Relative to lean non-NASH controls, obese NASH hamsters develop severe inflammation of the lungs and liver. 16S rRNA gene profiling showed that depending on the diet, SARS-CoV-2 infection induced various changes in the gut microbiota’s composition. Changes were more prominent and transient at day 4 post-infection in lean animals, alterations still persisted at day 10 in obese NASH animals. A targeted, quantitative metabolomic analysis revealed changes in the gut microbiota’s metabolic output, some of which were diet-specific and regulated over time. Our results showed that specifically diet-associated taxa are correlated with disease parameters. Correlations between infection variables and diet-associated taxa highlighted a number of potentially protective or harmful bacteria in SARS-CoV-2-infected hamsters. In particular, some taxa in obese NASH hamsters (*e.g. Blautia* and *Peptococcus*) were associated with pro-inflammatory parameters in both the lungs and the liver. These taxon profiles and their association with specific disease markers suggest that microbial patterns might influence COVID-19 outcomes.

## Introduction

Although vaccination against coronavirus disease 2019 (COVID-19) is now available in almost all countries, the COVID-19 pandemic is still a global health issue. In most individuals, infection by severe acute respiratory syndrome coronavirus 2 (SARS-CoV-2) is generally asymptomatic or induces only mild symptoms. However, the infection can lead to severe pneumonia, acute respiratory distress syndrome, multiple organ failure, and death in patients with risk factors such as old age and comorbidities.^1^,^2^ Among the later, obesity and nonalcoholic fatty liver diseases, including nonalcoholic steatohepatitis (NASH), are major risk factors of severe COVID-19.^3^,^4^ COVID-19 is a multisystem disease that ranges from respiratory tract manifestations to disorders in the intestines, liver, and heart. Given the gut-to-lung axis’ importance in health and disease and its critical role in immune responses and inflammation during respiratory tract infections,^5–8^ researchers have hypothesized that the gut microbiota has a critical role in COVID-19 (for reviews, see^9–11^). The gut microbiota is mostly composed of anaerobic bacteria. It is involved in many physiological functions, including digestion, metabolism, mucosal barrier integrity, organ functions, and immune homeostasis.^12^,^13^ The gut microbiota is altered in patients experiencing flu^14^,^15^ and during experimental influenza A virus infection (in a mouse model).^16–19^ Importantly, dysbiosis of the gut microbiota impacts disease outcomes.^18^,^20^ It is known that SARS-CoV-2 infection in experimental models (such as mice,^21^ hamsters^22^ and nonhuman primates^23^) also leads to gut dysbiosis. Accordingly, clinical studies highlighted that patients with severe COVID-19 have significant alterations in fecal microbiome, as characterized by the enrichment of potentially pathogenic bacteria and the depletion of beneficial commensals.^24–36^,^15^,^21^ Due to the moderate size of the cohorts studied, it is still not clear whether SARS-CoV-2 exacerbates preexisting gut dysbiosis in obese patients with NASH. Preclinical models capable of replicating the relevant metabolic features seen in humans have not previously been available.

The golden hamster replicates some of the metabolic features seen in humans – particularly in the case of underlying conditions such as dyslipidemia and NASH.^37–39^ The hamster has also recently emerged as an instrumental model of moderate, self-limiting, COVID-19.^40–47^,^22^ In a recent study, we found that SARS-CoV-2 infection in “healthy” hamsters (i.e. animals with normal physiological variables) led to a progressive alteration in the gut microbiota’s composition, with a higher relative abundance of harmful bacterial taxa (such as *Enterobacteriaceae* and *Desulfovibrionaceae*) and a lower relative abundance of short-chain fatty acid (SCFA) producers (such as *Ruminococcaceae* and *Lachnospiraceae* members).^22^ In the present study, we used a diet-induced model of obesity and NASH to assess the consequences of SARS-CoV-2 infection on the gut microbiota’s composition and functionality. We showed that SARS-CoV-2 infection in lean (non-NASH) hamsters and in obese NASH hamsters leads to different alterations in the gut microbiota’s composition and metabolic output. Interestingly, several infection-related variables were positively or negatively correlated with bacterial taxa found specifically in lean hamsters or in obese NASH hamsters. Some changes in the relative bacterial taxon abundance in obese NASH hamsters were specifically correlated with infection-related variables.

## Results

### During SARS-CoV-2-infection, levels of lung damage and liver inflammation are greater in obese NASH hamsters than in lean hamsters

**Figure 1.**
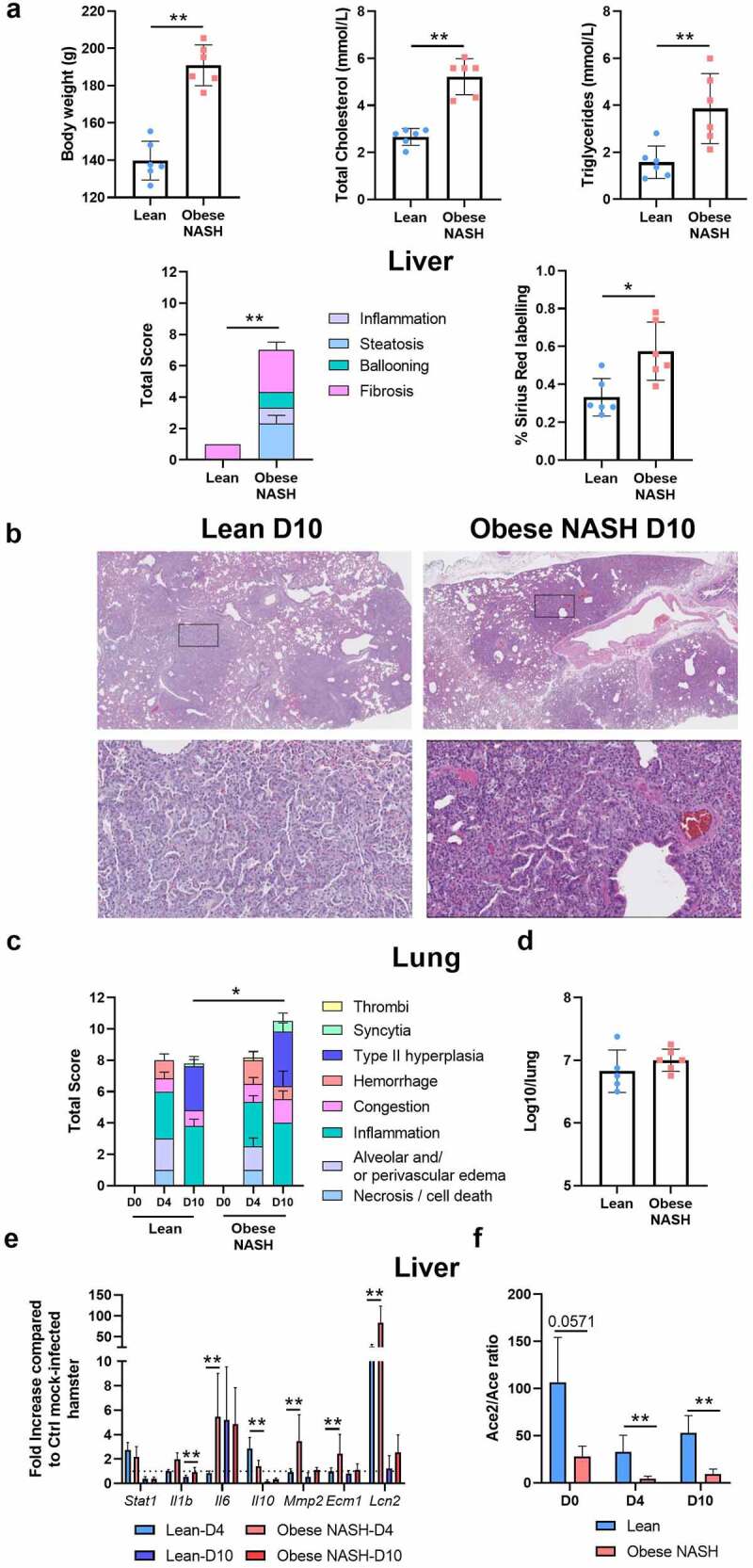
Establishment of a sublethal model of SARS-CoV-2 infection in obese NASH hamsters. (a), Clinical and biochemical parameters in lean and obese NASH hamsters. Body weight, total cholesterol and triglyceride concentrations in serum and liver pathology are shown. Total nonalcoholic fatty liver disease activity score and sirius red labeling score are depicted. (b), Lean and obese NASH hamsters were inoculated with 2 × 10^4^ tissue culture infectious dose 50 (TCID_50_) of the clinical SARS-CoV-2 isolate hCoV-19/France/lDF0372/2020. Histopathological examination of lung sections of SARS-CoV-2-infected hamsters lean and obese NASH hamsters (10 dpi). Representative images of lungs (hematoxylin and eosin staining) are depicted (x20). (c), Blinded sections were scored for levels of pathological severity. To evaluate comprehensive histological changes, lung tissue sections were scored based on criteria indicated in the panel. The following scoring system was used: 0, no pathological change; 1, affected area (≤10%); 2, affected area (<50%, >10%); 3, affected area (≥50%). The average sum of different parameters is shown. (d), Infectious viral loads in the lungs at D4. Data are expressed as the number of infectious virus particles per lung. At D10, no virus was detected in the lungs (not shown). (e and f), The liver of mock-infected and SARS-CoV-2-infected lean and obese NASH hamsters were collected at D4 and D10. mRNA copy numbers of genes were quantified by RT-PCR. Data are expressed as fold increase ± SD over average gene expression in mock-treated lean animals. (a-f), A representative experiment out of two is shown (n = 3–6/time point). Significant differences were determined using the Kruskal-Wallis ANOVA with Dunn’s posttest (*P < .05; ** *P* < .01; *** *P* < .001) .

Compared with hamsters fed a standard chow, hamsters fed a free-choice high fat/high cholesterol diet with drinking water enriched with 10% fructose for 20 weeks displayed higher body weight, suffered from dyslipidemia (*e.g*. higher serum levels of total cholesterol and triglycerides), and developed a substantial NASH and liver fibrosis phenotype (Figure 1a). We then investigated the effects of SARS-CoV-2 infection in lean hamsters and in free choice diet-induced obese NASH hamsters. Both groups showed a substantial reduction in body weight on post-infection day 7 (D7) (Supplementary Figure S1a) but started to recover thereafter (sacrifice at D10). With regard to lung disease, lean hamsters and obese NASH hamsters developed similar bronchointerstitial pneumonia at D4, together with bronchiolar epithelial cell death/necrosis, alveolar septal congestion, edema, and patchy alveolar hemorrhage (Supplementary Figure S1b). Lung inflammation was still severe at D10, and type II pneumocyte hyperplasia was clearly evidenced. Interestingly, lung lesions at D10 were more severe in obese NASH hamsters than in lean hamsters (Figure 1b and 1c).

A 50% tissue culture infectious dose (TCID50) assay showed that the infectious viral load in the lungs was similar in lean hamsters and obese NASH hamsters at D4 (Figure 1d). By D10, the TCID50 in the lungs had fallen to 0 (data not shown). An RT-PCR assay of viral RNA-dependent RNA polymerase (RdRp) levels confirmed the lack of major differences between lean hamsters and obese NASH hamsters at D4 (Supplementary Figure S1c). At D10, the viral load in lungs dropped dramatically and was slightly lower in obese NASH hamsters. Accordingly, the pulmonary virus-induced expression of genes related to interferons (IFNs) and interferon-stimulated genes (ISGs) (such as *Stat1, Mx1* and *Cxcl10*) was the same in lean hamsters and obese NASH hamsters (Supplementary Figure S1d). Expression of inflammation-related genes was either unchanged (*Il12p40, Ccl4, Cd4*,and *Cd11b*) or greater (*Il1b*) in obese NASH hamsters, relative to lean infected controls. Expression of genes related to barrier functions (*Zo1* and *cc10*) was similarly low during infection in the two groups (Supplementary Figure S1d).

It is known that SARS-CoV-2 infection in hamsters leads to extrapulmonary disorders, notably gut perturbation.^22^ Gene expression in the colon was altered at D4 and D10, albeit to the same extent in lean hamsters and obese NASH hamsters (Supplementary Figure S1e). In contrast, differences were observed in the liver. Although transcript levels for ISGs (*e.g. Stat1*) were not altered, the expression of various genes coding for inflammatory factors (*Il1b, Il6*) was enhanced in obese NASH hamsters, whereas the expression of genes coding for anti-inflammatory factors (*Il10*) was diminished (Figure 1e, the fold inductions, relative to mock-infected animals of the same diet, are depicted). Gene expression levels for *Mmp2* (coding for metalloproteinase 2), *Ecm1* (coding for extracellular matrix protein 1) and *Lcn2* (coding for lipocalin 2) were also higher in SARS-CoV-2-infected obese NASH animals than in infected lean controls (D4). It is noteworthy that the basal level of *Il6* and *Lcn2* mRNA expression was higher in obese NASH animals (Supplementary Figure S1 f). Activation of the hepatic renin-angiotensin system favors pro-inflammatory and pro-fibrotic profiles in mice fed a high fat diet.^48^ Angiotensin-converting enzyme 2 (ACE2) acts as a potent inhibitor of renin-angiotensin system activation.^49^ Accordingly, the D0 transcript ratio between ACE2 and angiotensin-converting enzyme (ACE, which activates the renin-angiotensin system) in the liver was dramatically lower in obese NASH hamsters than in lean hamsters (Figure 1f). SARS-CoV-2 infection led to a further relative reduction in the ACE2/ACE transcript ratio – particularly in obese NASH animals (D4 and D10). Overall, diet-induced obesity and NASH amplify lung damage and exacerbate the expression of liver inflammatory markers in SARS-CoV-2 infected hamsters.

### The changes in the composition of the gut microbiota associated with SARS-CoV-2 infection differ in lean hamsters vs. obese NASH hamsters

**Figure 2.**
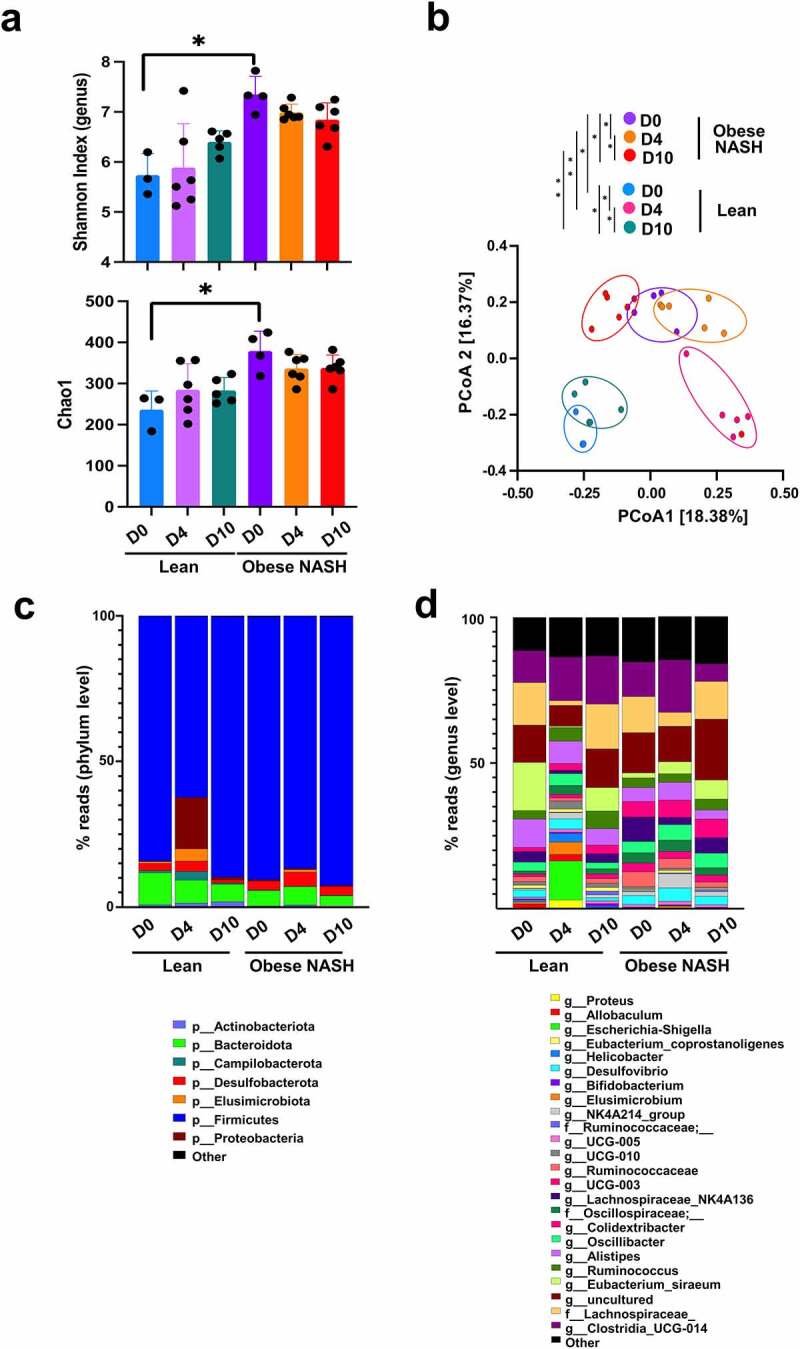
Changes over time of the gut microbiota’s composition during SARS-CoV-2 infection in lean and obese NASH hamsters. a, Shannon index and Chao1 index describing the α diversity of the bacterial microbiota in the various groups studied. Significant differences were determined using the Kruskal–Wallis ANOVA with Dunn’s posttest (*P < .05). b, Principal coordinate analysis of Bray-Curtis distance with each sample colored according to SARS-CoV-2 infection status and diet. PCoA1 and PCoA2 represent the top two principal coordinates that captured most of the diversity. The fraction of diversity captured by the coordinate is given as a percentage. Groups were compared using Permanova method (999 permutations). (c) and (d), Global composition of bacterial microbiota at the phylum (**c**) and genus (**d**) levels. Colored blocks indicate taxa with an average relative abundance. A representative experiment out of two is shown (n = 3–6/time point) (*P < .05) .

Next, we assessed and compared changes in the gut microbiota’s composition in lean animals vs. obese NASH animals at baseline and during SARS-CoV-2 infection (3 to 6 animals per group). To this end, feces samples from non-infected and infected hamsters were analyzed using 16S rRNA gene amplicon sequencing. We first analyzed bacterial diversity and richness, as measured by Shannon’s index and the Chao1 index, respectively (Figure 2a). At baseline (D0), obese NASH hamsters had higher α-diversity and richness relative to lean hamsters. Lean animals and obese NASH animals had distinct fecal microbiota compositions at D0, as shown by a β-diversity analysis based on the Bray Curtis distance (Figure 2b and Supplementary Figure S2a). Interestingly, infection led to a distinct shift in gut microbial composition, relative to baseline. In lean animals, intestinal communities were markedly disrupted at D4 and returned to the initial state at D10 (Figure 2b and Supplementary Figure S2b). Alteration of the gut microbiota at D4 was less intense in obese NASH animals than in lean animals. The Bray Curtis distance between lean hamsters and obese NASH hamsters fell at D4 and fell even more at D10 (Supplementary Figure S2a). This suggests that the effect of SARS-CoV-2 infection on the gut microbiota overrides that of the diet.

At the phylum level, the hamsters’ fecal microbiota was dominated by the Firmicutes (Figure 2c). Bacteroidota (Bacteroidetes), Desulfobacterota, Campilobacterota and (albeit to a lesser extent) Elusimicrobiota were also detected. At D0, the lean hamsters and obese NASH hamsters differed with regard to the taxonomic composition of the gut microbiota (Figure 2c and Supplementary Figure S2c). The relative abundance of several phyla changed during SARS-CoV-2 infection. It is noteworthy that the relative abundance of the Elusimicrobiota, Campilobacterota, and particularly Proteobacteria increased at D4 in lean hamsters (at the expense of the Firmicutes) (Figure 2c and Supplementary Figure S2c). A significant impact of infection on lower taxonomic levels was also observed (Figure 2d).

### Diet and SARS-CoV-2 infection affect the gut microbiota’s composition in different ways

**Figure 3.**
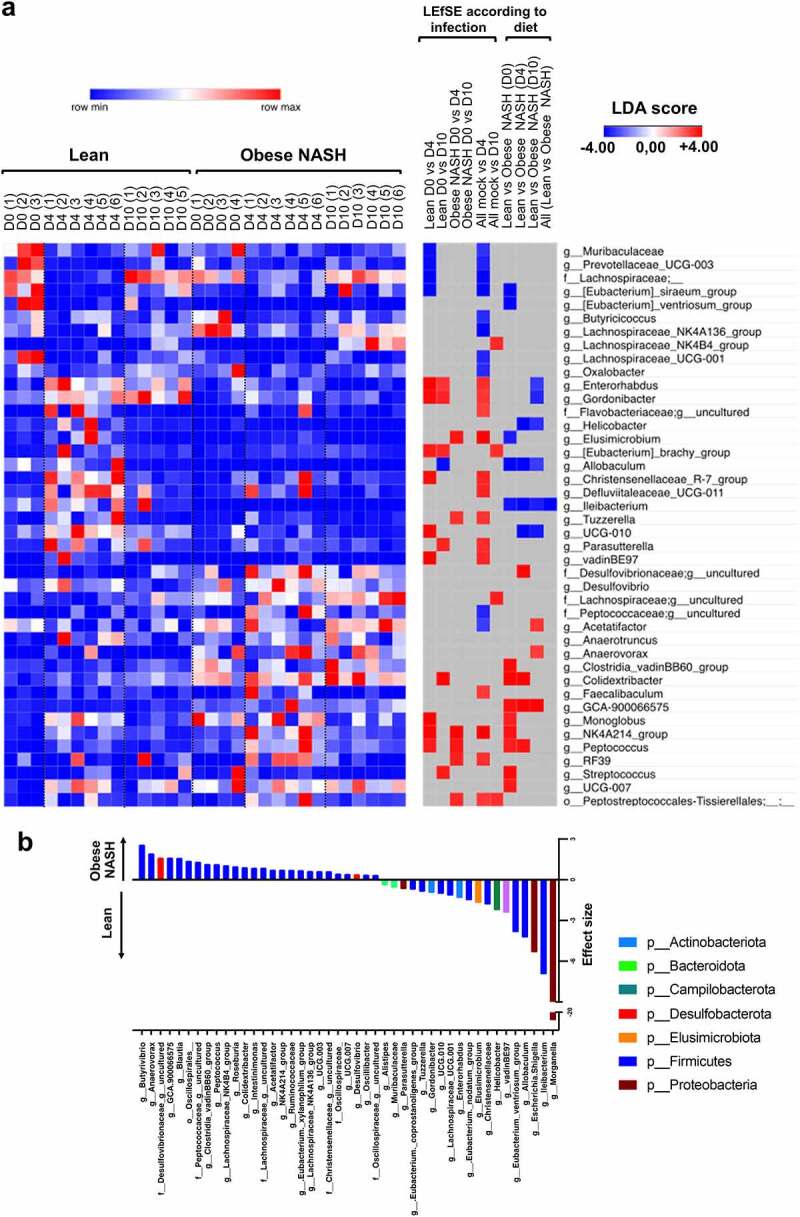
Alterations in the fecal microbiota’s composition over the course of a SARS-CoV-2 infection in lean and obese hamsters. a, A linear discriminant analysis effect size (LEfSe) analysis was performed to represent bacterial taxa changed over the course of the infection according to the diet of the animals. Only taxa with a statistically significant LDA score (log10) > 2 (compared with mock and/or compared to normal diet) are shown. The heat map on the *left* panel shows the relative abundance of the taxa, and the heat map on the right shows the LDA scores. The taxa are clustered by abundance pattern by day and diet using K-mean clustering (one minus cosine similarity). **b**, Maaslin2 analysis at the genus level of fecal bacteria associated with lean/NASH obese conditions adjusted for SARS-CoV-2 infection status.

The gut microbiota’s composition at the family and genus levels changed significantly over time, as shown by linear discriminant analysis effect size (Figure 3a). Most of the significant changes in the microbiota at D4 were transient, except for those affecting the genera *Enterorhabdus* and *Gordonibacter* (in lean hamsters) and *Desulfovibrio* and *Anaerovax* (in obese NASH hamsters); the latter were still present at D10. In obese NASH hamsters, some species (such as *Lachnospiraceae_NK4A136* and *Acetatifactor*) were only altered at D10. With an adjustment for diet, a microbiome multivariable association with linear models (MaAsLin2) analysis showed that SARS-CoV-2 infection was associated with specific changes in proinflammatory taxa, such as the Gammaproteobacteria members *Morganella* and *Escherichia-Shigella* (Supplementary Figure S3a). These results are in line with our previous findings.^22^ In infected animals, we observed a lower relative abundance of several SCFA-producing Firmicutes (the Clostridia class, known to contain important butyrate-producing species), including *Lachnospiraceae_NK4A136* (mostly in obese NASH animals), *Lachnospiraceae_UCG-001* (in lean animals), and *Butyricicoccus* (Figure 3a and Supplementary Figure S3a). In lean hamsters, the abundance of the acetate-producing bacterium *Eubacterium siraeum* (*Ruminococcaceae*) fell during the SARS-CoV-2 infection (Figure 3a).

Some differences in taxons at basal level between lean hamsters or obese NASH hamsters were still present during infection. These include *Ileibacterium* (*Erysipelotrichaceae*) in lean hamsters and *GCA-900066575* (*Lachnospiracaea*) and *Colidextribacter* (*Oscillospiracea*) in obese NASH hamsters (but not at D10 for the latter) (Figure 3a and Supplementary Figure S3b). In contrast, some taxa only differed during the infection: these include *UCG_010, Helicobacter, Enterorhabdus* and *Gordonibacter* (enhanced in lean hamsters during the infection) and *Desulfovibrio, Acetatifactor*, and *Anaerovorax* (enhanced in obese NASH hamsters during the infection) (Figure 3a and Supplementary Figure S3b). A multivariate analysis performed with MaAsLin2 to identify taxa associated with each diet and adjusted for infection status showed similar results (Figure 3b). *Morganella, Ileibacterium* and *Escherichia*/*Shigella* were the top three taxa associated with the lean condition, whereas *Butyrivibrio, Anaerovorax* and *Desulfovibrionaceae* were the top three associated with the obese NASH condition (Figure 3b). Hence, diet and SARS-CoV-2 infection affect the gut microbiota’s composition in different ways.

### The changes in the gut microbiota’s metabolic output associated with SARS-CoV-2 infection differ in lean hamsters vs. obese NASH hamsters

**Figure 4.**
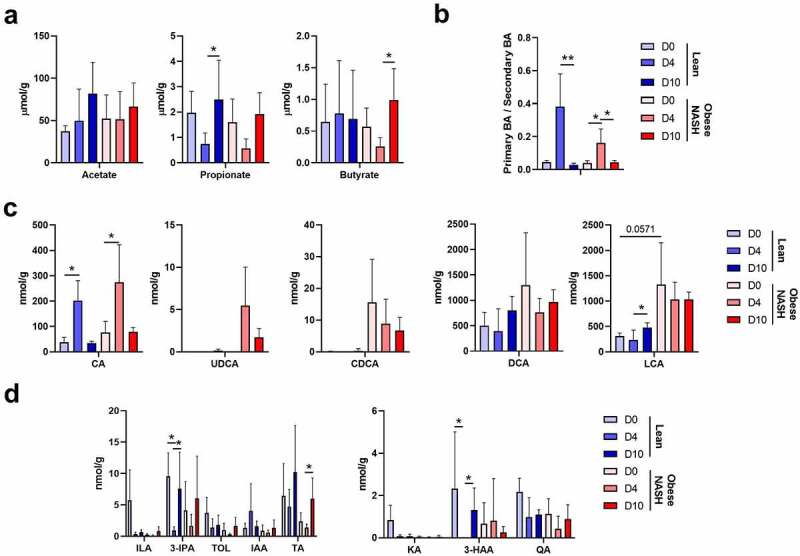
Alteration in fecal metabolite production during a SARS-CoV-2 infection. SCFAs (a), BAs (b and c) and tryptophan metabolites (d) were measured in fecal samples from each animal and at each time point, using targeted quantitative metabolomics. Values for individual animals are presented. CA, cholic acid; CDCA, chenodeoxycholic acid; DCA, deoxycholic acid; LCA, lithocholic acid; UDCA, ursodeoxycholic acid; ILA, indole-3-lactic acid; 3-IPA, 3-Indole propionic acid; TOL, indole-3-ethanol; IAA, indole-3-acetic acid; TA, tryptamine KA, kynurenic acid; 3-HAA, 3-hydroxyanthranilic Acid; QA, quinolinic acid. A representative experiment out of two is shown (n = 3–6/time point). Significant differences were determined using the Kruskal–Wallis ANOVA with Dunn’s posttest (*P < .05). To compare values between lean and obese NASH hamsters at D0, a student t test was used.

To evaluate the functional consequences of a SARS-CoV-2 infection, we quantified fecal levels of three of the most important categories of microbiota-derived metabolites namely SCFAs, bile acids (BAs), and tryptophan metabolites.^50^ The fermentation products SCFAs have a key role in intestinal barrier, immune and metabolic functions.^50–53^ Basal concentrations of SCFAs were similar in non-infected lean hamsters and non-infected obese NASH hamsters (Figure 4a). The fecal level of acetate did not change during the infection. In contrast, propionate levels decreased transiently at D4 in both groups of animals, while those of butyrate fell only in obese NASH hamsters. Interestingly, low butyrate levels are associated with systemic inflammation and disease severity in COVID-19 patients.^28^

Primary BAs (produced by the liver as conjugates) are transformed into secondary BAs by the gut microbiota. Secondary BAs have important functions within and outside the gastrointestinal tract notably through their action on nuclear (farnesoid X receptor) and transmembrane (Takeda G-protein receptor *5*) receptors.^50^,^52^ They play an important role in the modulation of epithelial cell proliferation, gene expression and lipid and glucose metabolism. The primary to secondary BA ratio at D0 was similar in lean hamsters and obese NASH hamsters (Figure 4b). Interestingly, SARS-CoV-2 infection was associated with a large, transient increase in this ratio in both lean hamsters and obese NASH hamsters, indicating an impairment in BA transformation by the gut microbiota during SARS-CoV-2 infection. The increase of primary BAs at D4 involved cholic acid (CA, in lean and obese hamsters) and ursodeoxycholic acid (UDCA, in obese NASH hamsters) but not chenodeoxycholic acid (CDCA) (Figure 4c). The levels of the secondary BAs deoxycholic acid (DCA) and lithocholic acid (LCA) decreased in lean and obese NASH hamsters without reaching statistical significance.

Gut cells can metabolize tryptophan into various compounds, via the serotonin and indoleamine 2,3-dioxygenase pathways. Tryptophan is also metabolized into indoles by gut bacteria; this is an important process for intestinal homeostasis because indoles can activate the aryl hydrocarbon receptor. The latter regulates gut barrier permeability, antimicrobial peptide secretion and mucosal immunity.^53^,^54^ At D0, the overall concentration of tryptophan metabolites was lower in obese NASH hamsters than in lean hamsters (Figure 4d). This finding is in line with preclinical and clinical studies in a metabolic syndrome context, in which the gut microbiota produced smaller amounts of aryl hydrocarbon receptor agonists.^55^ Changes in the abundance of several tryptophan metabolites were observed in SARS-CoV-2-infected hamsters. In particular, levels of indole-3-lactic acid (ILA) and 3-indole propionic acid (3-IPA, known to be decreased during mucosal inflammation^53^,^56^) fell dramatically at D4 – especially in lean animals (Figure 4d, *left* panel). We also observed a decreased relative abundance of metabolites from the indoleamine 2,3-dioxygenase pathway including kynurenic acid (KA), 3-hydroxyanthranilic acid (3-HAA) and, to a lesser extent, the end product quinolinic acid (QA), mostly in lean animals (Figure 4d, *right* panel). Taken as a whole, the results of this targeted approach showed that diet influences functional changes in the gut microbiota during a SARS-Cov-2 infection.

### Correlation between infection variables and diet-associated taxa reveals potentially protective and harmful bacteria in SARS-CoV-2-infected hamsters

We then sought to determine whether changes of gut microbiota’s composition (whatever the diet) were correlated with infection parameters, including lung and liver inflammation. Taxa associated with a pro-inflammatory signature were similar to what we and others already described^21^,^22^ (Supplementary Figure S4). The abundance of *Elusimicrobium, Oscillospiraceae* and members of the Proteobacteria Phylum (such as the genus *Desulfovibrio*) were positively correlated with pro-inflammatory cytokine levels. Genus that correlate with a high and low histological score of inflammation in the lung were those with the strongest association with obese NASH hamsters and lean hamsters, respectively (Figure 3b, indicated by an arrow in Supplementary Figure S4).

**Figure 5.**
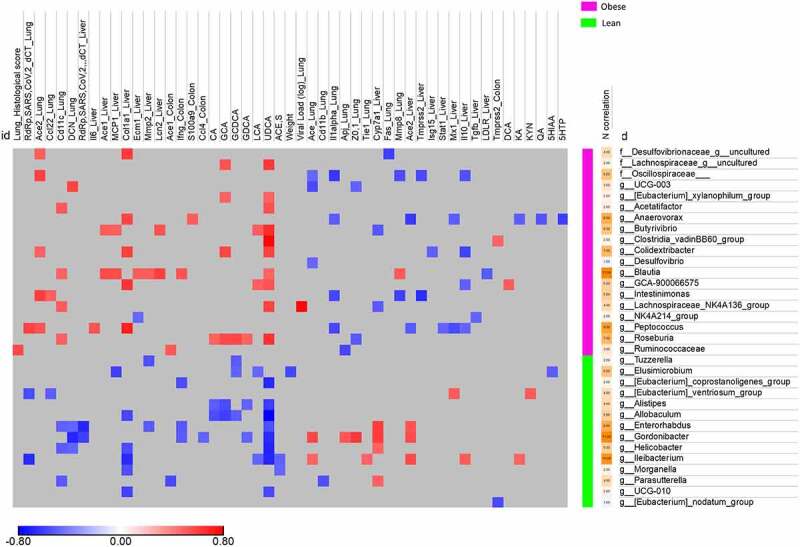
Correlation between diet-associated bacterial taxa and infection-related variables. Heatmap of Spearman coefficient between SARS-CoV-2 infection related variables and taxa at the genus level selected from the MaAsLin2 analysis of Figure 3b. Taxa and SARS-CoV-2 infection related variables are clustered by diet-specificity. Only significant correlations (p < .05 and q < 0.25 after correction for the false discovery rate, using the Benjamini-Hochberg procedure) are shown.

We next looked at whether any of the specific, diet-associated taxa identified in the MaAsLin2 analysis (Figure 3b) were significantly correlated with SARS-CoV-2 infection variables. Twenty-six bacterial species were associated with various categories of infection variables, and almost all were associated with three or more categories (Figure 5). Interestingly, when taxa were clustering according to the diet, the obese NASH-associated taxa were positively correlated with (i) greater viral RNA loads in the lung and the liver and (ii) pro-inflammatory marker profiles in the lung (the histology score and mRNA levels of inflammation-associated factors), the colon (mRNA levels of *Ifng* and *S100a9*) and the liver (mRNA levels of pro-fibrotic factors such as *Col1a1, mmp2, Ecm1* and *Lcn2*). In contrast, lean-associated taxa were less tightly correlated with inflammatory and pro-fibrotic profiles.

Some species were correlated with a large number of SARS-CoV-2-associated variables. For instance, *Blautia* and *Peptoccocus* (observed predominantly in obese NASH hamsters) were correlated with a large number of pro-inflammatory or pro-fibrotic factors profiles (and, for *Peptoccocus*, the viral load in the lung). In contrast, *Gordonibacter* and *Ileibacterium* (observed predominantly in lean animals) were negatively correlated with inflammatory profiles (and, for *Ileibacterium*, the viral load in the lung) (Figure 5). We conclude that several parameters linked to infection were positively or negatively correlated with bacterial taxa found specifically in lean hamsters or obese NASH hamsters; in turn, these correlations suggest that diet-induced alterations in the microbiota are associated with the severity of infection.

## Discussion

Co-morbidities like obesity and nonalcoholic fatty liver diseases strongly influence the outcome of COVID-19. In this context, changes in the gut microbiota during infection might provide important information about pathological mechanisms and disease outcomes.^10^ Clinical studies have highlighted changes in the composition of the gut microbiota during SARS-CoV-2 infection, although specific data for obese individuals and patients with nonalcoholic fatty liver diseases are still lacking.^24–36^,^15^,^21^ Although these clinical studies are essential for translational purposes, they have several limitations. For instance, with regard to the gut microbiota, it is difficult to distinguish between the direct effects of viral infection on one hand and the impact of hospitalization and medical treatment on the other. The development of animal models of human disease is a complementary approach that (if validated) might reveal the functional significance of changes in the gut microbiota. The pathophysiologic similarities between humans and golden hamsters mean that the latter is a useful model of COVID-19.^40–47^,^22^ Our recent study on healthy adult hamsters showed that SARS-CoV-2 infection led to changes in the gut microbiota’s composition and that these changes were correlated with disease severity.^22^ In the present study, we sought to determine the impact of a SARS-CoV-2 infection in a hamster model of obesity and NASH. Our results suggest that obesity and NASH accentuate inflammation in the lung and liver during a SARS-CoV-2 infection. This nutritional model might be of value in determining impacts on the gut microbiota‘s composition and metabolic outputs.

The gut microbiome baseline’s configuration is likely to be important for COVID-19 outcomes; an emerging body of evidence indicates that changes in the microbiome might predict the development of specific acute and post-acute signs and symptoms of COVID-19.^31–33^,^25^,^27^,^28^,^36^ In line with the literature data,^57^,^58^ our β-diversity analysis and taxonomic description showed that lean hamsters and obese NASH hamsters had distinct fecal microbiota compositions at baseline. The recent literature suggests that the gut microbiota’s composition and metabolic activity are important for the control of respiratory viral infections.^8^,^59^,^60^ In our settings, differences in the gut microbiota configuration between lean hamsters and obese NASH hamsters did not influence the viral load in lungs but were associated with greater lung damage and liver inflammation. The infection’s impact on the gut microbiota’s composition differed according to the diet. Surprisingly, we found that the changes were more intense in lean hamsters than in obese NASH hamsters at D4. The Proteobacteria (*Escherichia-Shigella, Parasuturella*), Elusimicrobiota (*Elusimicrobium*) and Firmicutes were particularly impacted. Overall, the composition of the gut microbiota returned to the basal profile at D10, although some differences still persisted (*Enterorhabdus, Gordonibacter*, and *Eleibacterium*). In obese NASH hamsters, changes at D4 were less marked but some persisted at D10 (*Lachnospiraceae NK4B4_group, Desulfovibrionaceae_uncultured, Acetatifactor*, and *Monoglobus*). Hence, the infection’s impact on the composition of the gut microbiota is different according to the diet.

The changes in the gut microbiota’s composition during SARS-CoV-2 infection translated into several metabolic changes. The SCFA levels changed during SARS-CoV-2 infection in both lean hamsters and obese NASH hamsters. In line with our previous findings,^22^ we did not see significant changes in acetate levels. In contrast, propionate levels diminished transiently during infection (in both groups), as did butyrate levels (in obese NASH hamsters). Importantly, the drop in butyrate is reportedly correlated with systemic inflammatory marker levels and the severity of COVID-19 in humans.^28^ To our surprise, the drops in propionate and butyrate levels were not significantly associated with changes in bacterial species (Figure 5), although we observed a change in the abundance of putative SCFA producers during the infection. Bile acid metabolism (DCA and LCA) and the production of tryptophan metabolites (ILA, 3-IPA, KA and 3-HAA) were also affected, with notable differences between the lean and obese NASH groups. Given the key role of SCFAs, secondary BAs and tryptophan metabolites in the host’s immune homeostasis, these perturbations are likely to have major consequences for systemic and local inflammation. Further functional analyses will be required to establish a direct causal relationship between changes in the gut microbiota, changes in metabolite levels, and disease severity in this infectious model. It would also be useful to investigate the significance of the difference in the changes in taxa abundance and metabolite concentrations between lean hamsters (with an apparent peak at D4) and obese NASH hamsters (without a peak).

Our study is novel in that we assessed severity markers and their associations with the altered gut microbiota in hamsters on two different diets. However, the study also had some limitations. Under our experimental conditions, SARS-CoV-2-infected obese NASH hamsters did not develop severe COVID-19 (*e.g*. organ failure and death). Hence, the obese NASH hamster model is less relevant – at least at the time points analyzed here – with regard to the human disease. In the future, it will be interesting to integrate other indicators of disease outcomes such as organ functions. The fact that obese NASH hamsters take longer to recover their pre-infection body weight (data not shown) suggests that it would be worth studying the long-term consequences of SARS-CoV-2-infection in these animals. Long-lasting effects on the gut microbiota’s composition (>6 months) have been described in COVID-19 patients.^31–33^,^25^,^27^,^28^,^36^ As some bacterial taxa and the associated metabolites are linked to long-term sequela,^25^,^27^,^28^ it will be necessary to study later time points. Metabolism, liver function, cardiac functions, pulmonary fibrosis and hepatic fibrosis might potentially be correlated with gut microbiota changes in obese NASH hamsters. Despite these limitations, our study is the first to have described gut microbiota changes in a hamster model of obesity and NASH. Our results showed that specifically diet-associated taxa are correlated with disease parameters. Some changes in relative bacterial taxon abundances were specifically correlated with infection variables in obese NASH hamsters. In particular, some taxa in obese NASH hamsters (*e.g. Blautia* and *Peptococcus*) were associated with pro-inflammatory parameters in both the lung and the liver. These taxon profiles and their association with specific disease markers suggest that microbial patterns might influence COVID-19 outcomes. Obese NASH hamsters might be of value in designing gut-microbiota-targeting treatments for COVID-19.

## Materials and methods

### Animals and ethics

All animal protocols were reviewed and approved by the local ethics committee (Comité régional d’éthique de Midi-Pyrénées, CEEA-122–2014-15 and APAFIS#23428-2019070815255735) and Comité d’Ethique en Expérimentation Animale (CEEA) Nord/Pas-de-Calais 75 and national ethics committee (ministère de l’Enseignement Supérieur et de la Recherche) (Protocol #CEEA-122–2014-15 and APAFIS#25041-2020040917227851 v3). After a 5-day acclimation period, male Golden Syrian hamsters (4-week-old at the beginning of the study) (Janvier Labs, Le Genest-St-Isle, France) were fed for up to 20 weeks either a control chow diet (5.1% fat, 19.3% protein, 55.5% carbohydrates, from SAFE Diets, Augy, France) with access to regular laboratory animal drinking water, or a free-choice diet. The free-choice diet consists of a choice, within the same cage, between control chow diet with regular drinking water or a high fat/high cholesterol diet (40.8% fat, 14.8% protein, 44.4% carbohydrates and 0.5% cholesterol from SAFE Diets) with 10% fructose-enriched drinking water. The high fat/high cholesterol diet consists in a mixture of 55% control chow diet, 20% peanut butter paste (Skippy, Hormel Foods Corporation, Austin, MN, USA) and 25% hazelnut paste (Nustikao, Leclerc, Ivry-sur-Seine, France), with vegetable oils as fat source, as described previously.^61^ After a 2-day recovery period, hamsters were fasted for 6 hours and blood was collected by retro-orbital sampling under isoflurane anesthesia for biochemistry analyses on serum. Animals were then euthanized and exsanguinated with saline prior to organ collection (lung and liver) for biochemistry and histology analyses.

Another set of lean and obese NASH hamsters fed a chow or free-choice diet for 20 weeks were transferred to the Institut Pasteur de Lille biosafety level 3 (BSL3) facility for SARS-CoV-2 infection. All SARS-CoV-2 infection experiments complied with current national and institutional regulations and ethical guidelines (Institut Pasteur de Lille/B59-350009). After 7 days acclimation, animals were infected intranasally with a sub-lethal dose of the SARS-CoV-2 clinical isolate BetaCoV/France/IDF/0372/2020 strain, supplied by the French National Reference Center for Respiratory Viruses hosted by Institut Pasteur (Paris, France).^22^ The virus was propagated in Vero-E6 cells (ATCC number CRL-1586) expressing TMPRSS2 by inoculation at MOI 0.01. Cell supernatant medium was harvested at 72 h post-infection and stored frozen at −80°C in small aliquots (2 x 10^6^ TCID_50_/mL). All experiments were conducted in a biosafety level 3 laboratory (BSL3) laboratory. For infection, hamsters were anesthetized by intraperitoneal injection of 300 µl containing ketamine (100 mg/kg), atropine (0.75 mg/kg) and valium (2.5 mg/kg) and then infected intranasally with 100 µl of DMEM without (mock hamsters) or with SARS-CoV-2 viruses (2x10^4^ TCID_50_). Body weight was monitored before and after SARS-CoV-2 infection. All animals were infected and kept in isolators within the BSL3 facility. For tissue collection, animals were euthanized by intraperitoneal injection of euthasol at 140 mg/kg. Blood, lungs, and liver were collected from non-infected (mock) hamsters and from SARS-CoV-2-infected hamsters 4 and 10 days after infection (3 to 6 animals per group). For ethical concerns, different parameters were analyzed on the same lung. The two right lobes of the lung were used to quantify the viral load and the other two right lobes were used for gene expression. The left lobe was kept for histology. This procedure is included in the limitation of the study.

### Reagents

Methanol (MeOH), acetonitrile and formic acid were of HPLC grade. Analytical grade of NaOH, propan-1-ol, pyridine, hexane and propylchloroformate (PCF) were purchased as well from Sigma-Aldrich (Saint Quentin Fallavier, France). Deionized water comes from a Milli-Q Elix system fitted with a LC-PaK and a MilliPak filter at 0.22 μm (Merck Millipore, Guyancourt, France). The following bile acid standards: cholic acid (CA), chenodeoxycholic acid (CDCA), deoxycholic acid (DCA), lithocholic acid (LCA), ursodeoxycholic acid (UDCA), hyodeoxycholic acid (HDCA), lycocholic acid (GCA), glycochenodeoxycholic acid (GCDCA), glycodeoxycholic acid (GDCA), glycolithocholic acid (GLCA), taurocholic acid (TCA), taurochenodeoxycholic acid (TCDCA), taurodeoxycholic acid (TDCA), tauroursodeoxycholic acid (TUDCA), and taurolithocholic acid (TLCA) and ammonium acetate were purchased from Sigma Chemical (St. Louis, MO, USA). All tryptophan reference, isotope labeled metabolites and SCFAs (acetate, propionate, butyrate, isobutyrate, valerate, isovalerate, acetate-D3, butyrate-13C2 and valerate-D9) were purchased from Sigma-Aldrich (Saint Quentin Fallavier, France). The stock solutions of bile acids, tryptophan metabolite and SCFAs were prepared separately in methanol at the concentration of 10 mmol/l.

### Determination of viral load and gene expression levels by quantitative RT-PCR

Infectious virus load and viral RNA were determined by using the Reed & Muench tissue culture infectious dose 50 (TCID_50_) assay and quantitative reverse transcription PCR (RT-qPCR), respectively. For titration of live infectious virus, half of right lobes were homogenized in Lysing Matrix D tubes containing 1 ml of PBS using the Mixer Mill MM 400 device (Retsch) (15 min – 15 Hz). After centrifugation at 11,000 rpm for 5 min, the clarified supernatant was harvested for live virus titration. Dilutions of the supernatants were done in DMEM with 1% penicillin/streptomycin and dilutions were transferred to Vero-E6 cells in 96-well plates for TCID50 assay. Briefly, serial 10-fold dilutions of each sample were inoculated in a Vero-E6 cell monolayer in duplicate and cultured in DMEM supplemented with 2% fetal bovine serum (Invitrogen, Waltham, MA) and 1% penicillin/streptomycin and L-glutamine. The plates were observed for cytopathic effects for 5–6 days. Virus titers were expressed as TCID_50_ corresponding to the amount of virus that caused cytopathic effects in 50% of inoculated wells. For viral RNA quantitation in lung tissue, half of the right lobe was homogenized in 1 ml of RA1 buffer (NucleoSpin RNA kit, Macherey Nagel) containing 20 mM of Tris(2-carboxyethyl)phosphine hydrochloride (TCEP). Total RNAs in the tissue homogenate were extracted using the NucleoSpin RNA kit. RNA was reverse-transcribed with the High-Capacity cDNA Archive Kit (Life Technologies, USA). The resulting cDNA was amplified using SYBR Green-based real-time PCR and the QuantStudio™ 12 K Flex Real-Time PCR Systems (Applied Biosystems™, USA) following manufacturers protocol. Relative quantification was performed using the gene coding RNA-dependent RNA polymerase (*RdRp*) and glyceraldehyde 3-phosphate dehydrogenase (*gapdh*). Analyses of gene expression in lungs, colon and liver were performed by classical procedures. Specific primers were designed using Primer Express software (Applied Biosystems, Villebon-sur-Yvette, France) and ordered to Eurofins Scientifics (Ebersberg, Germany) (Table 1). The list of primers is available in Table 1. Relative mRNA levels (2^−ΔΔCt^) were determined by comparing (a) the PCR cycle thresholds (Ct) for the gene of interest and the house keeping gene (ΔCt) and (b) ΔCt values for treated and control groups (ΔΔCt). Data were normalized against expression of the γ-actin and are expressed as a fold-increase over the mean gene expression level in mock-treated mice. Viral load is expressed as viral RNA normalized to γ-actin expression level (ΔCt).

**Table 1.** Oligonucleotide sequences used in this study

*y-actin*	F 5’-ACAGAGAGAAGATGACGCAGATAATG-3’	*Il10*	F 5’-GGTTGCCAAACCTTATCAGAAATG-3’
	R 5’-GCCTGAATGGCCACGTACA-3’		R 5’-TTCACCTGTTCCACAGCCTTG-3’
*RdRp SARS-CoV-2*	F 5’-GTGARATGGTCATGTGTGGCGG-3’	*Il12p40*	F 5’-AATGCGAGGCAGCAAATTACTC-3’
	R 5’-CARATGTTAAASACACTATTAGCATA-3’		R 5’-CTGCTCTTGACGTTGAACTTCAAG-3’
*Ccl4*	F 5’-CTCTGCCATGCTTTTGTGCC-3’	*Lcn2*	F 5’-CCAACCAGCCATTGATCCCT-3’
	R 5’-ATCAGCCCATCTCACCACAG-3’		R 5’-TCACAACGTTGGTCCCTGAG-3’
*Cc10*	F 5’-ACAAGCCCTCTGTGCAATCA-3’	*Mmp2*	F 5’-CTCTCGAATCCATGACGGGG-3’
	R 5’-GGGGCTGTTATCAGGGAGTG-3’		R 5’-AACACCAGAGGAAGCCATCG-3’
*Cd11b*	F 5’-CCCGTCGAGAGCTTGATACC-3’	*Muc4*	F 5’-GGAAAACGAAAACGCCTCCC-3’
	R 5’-AGTGTGCTGATATCGAGGCG-3’		R 5’-AAAATGGATGACCGGACCCC-3’
*Cd11c*	F 5’-GAGGCCTCTGCTCTTCACTC-3’	*Mx1*	F 5’-GGTATCGTTACCAGGTGCCC-3’
	R 5’-AAGAAACAAACGCCGTCAGC-3’		R 5’-GGTCTGGAACACTTGGGGAG-3’
*Cd4*	F 5’-TCCCACTCCGCCTCAAGATA-3’	*Mx2*	F 5’-CCAGTAATGTGGACATTGCC-3’
	R 5’-TGGCGCCGTTGGTGTC-3’		R 5’-CATCAACGACCTTGTCTTCAGTA-3’
*Cxcl10*	F 5’-TACGTCGGCCTATGGCTACT-3’	*Stat1*	F 5’-TCCATGCGGTTGAACCCTAC-3’
	R 5’-TTGGGGACTCTTGTCACTGG-3’		R 5’-TGTCAGTGTTCTGTGCTCACTT-3’
*Ecm1*	F 5’-TGGGGACCATATCCAGAGCA-3’	*S100a8*	F 5’-ACTGCTCACGACTGAGTGTC-3’
	R 5’-GGCTTCATCTCTCTCGGCTC-3’		R 5’-GCCAGGCCCACCTTTATCAT-3’
*Il1b*	F 5’-GAAGTCAAAACCAAGGTGGAGTTT-3’	*S100a9*	F 5’-CACGAGTCTAGCAAGGGACA-3’
	R 5’-TCTGCTTGAGAGGTGCTGATGT-3’		R 5’-TGGTTTCTATGCTGCGCTCC-3’
*Il6*	F 5’-CCATGAGGTCTACTCGGCAAA-3’	*Zo1*	F 5’-CTCCTGCCGCTCAAAAGGA-3’
	R 5’-GACCACAGTGAATGTCCACAGATC-3’		R 5’-CGCCGGAAGTAGCACCATTA-3’

### Histopathology

Liver histology (H&E and Sirius Red staining), NAFLD activity scoring and % Sirius Red labeling were performed as described previously.^62^ Lung tissues were fixed in 4% PBS buffered formaldehyde for 7 days, rinsed in PBS, transferred in ethanol (70%) and then processed into paraffin-embedded tissues blocks. The subcontractor Sciempath Labo (Larçay, France) performed histological processing and analysis. Tissue sections (3 µm) were stained with hematoxylin and eosin (H&E) and whole mount tissues were scanned with a Nanozoomer (Hamatsu) and the morphological changes were assessed by a semi-quantitative score. For the scoring, a dual histopathology scoring system adapted from^42^,^63^ was used to assess pulmonary changes in hamsters. A total of nine parameters was qualitatively assessed and ranked with a score from 0 to 4: (1) cellular death/necrosis, (2) alveolar and/or perivascular edema, (3) hyaline membrane or fibrin, (4) inflammation, (5) thrombi, (6) congestion, (7) hemorrhage, (8) type II hyperplasia, and (9) syncytia. For each criteria, a score 0 = absent, 1 = 1–10% of lung section, 2 = 11–25% of lung section, 3 = 26–50% of lung section, and 4 = >50% of lung section affected.

### Sample collection, genomic DNA extraction and sequencing

To study the impact of SARS-CoV-2 infection on gut microbiota, hamsters were intranasally infected and their feces were collected at D4 and D10. Feces from mock-infected hamsters served as controls (D0). Fresh fecal samples were stored at −80°C until further analyses. Microbial DNA was extracted from 200 mg of fecal samples as previously described.^54^ Following microbial lysis with both mechanical and chemical steps, nucleic acids were precipitated in isopropanol for 10 minutes at room temperature, incubated for 15 minutes on ice and centrifuged for 30 minutes at 15,000 *g* and 4°C. Pellets were resuspended in 112 µl of phosphate buffer and 12 µl of potassium acetate. After RNase treatment and DNA precipitation, nucleic acids were recovered via centrifugation at 15,000 *g* and 4°C for 30 minutes. The DNA pellet was resuspended in 100 µl of TE buffer. The concentration of extracted DNA was determined using on a DNA fluorometric intercalant (SYBR® Green, ThermoFisher Scientific (Waltham, MA). Microbial diversity and composition were determined for each sample by targeting a portion of the ribosomal genes. A 16S rRNA gene fragment comprising V3 and V4 hypervariable regions (16S; 5′-TACGGRAGGCAGCAG-3′ and 5′-CTACCNGGGTATCTAAT-3′) was amplified using an optimized and standardized 16S-amplicon-library preparation protocol (Metabiote, GenoScreen, Lille, France). Briefly, 16S rRNA gene PCR was performed using 5 ng genomic DNA according to the manufacturer’s protocol (Metabiote) using 192 bar-coded primers (Metabiote MiSeq Primers, GenoScreen) at final concentrations of 0.2 μM and an annealing temperature of 50°C for 30 cycles. The PCR products were purified using an Agencourt AMPure XP-PCR Purification system (Beckman Coulter), quantified according to the manufacturer’s protocol, and multiplexed at equal concentrations. Sequencing was performed using a 250-bp paired-end sequencing protocol on an Illumina MiSeq platform (Illumina) at GenoScreen. Positive (artificial bacteria community comprising 17 different bacteria (ABCv2)) and negative (sterile water) control were also included.

### Gut microbiota analysis

Following DNA extraction and sequencing, raw paired-end reads were processed in a data curation pipeline that includes a step of removal of low quality reads (Qiime2 2020.8). Remaining sequences were assigned to samples based on barcode matches, and barcode and primer sequences were then trimmed. The sequences were denoized using the DADA2 method, and reads were classified using Silva reference database (version 138). A total of 1,212,603 sequence reads were analyzed, with an average of 39,116 per sample (range: 20,242 to 53,166). Alpha and beta diversity were computed using Qiime2 2020.8. Principal Coordinate analyses of the Bray Curtis distance were performed to assess beta diversity. Chao1 and Shannon indexes were calculated to characterize alpha diversity. Raw sequence data are accessible in the National Center for Biotechnology Information (project number PRJNA800738), biosample accession numbers SAMN25277727 to SAMN25277756. Differential analysis was performed using the linear discriminant analysis effect size (LEfSe) pipeline and MaAsLin2 package (version 1.7.2). Spearman’s correlations between bacterial taxa and SARS-CoV-2 infection parameters were analyzed. Correlations were considered significant when *P* values < .05 with q < 0.25 after correction for the false discovery rate, using the Benjamini-Hochberg procedure.

### Metabolomic analysis of fecal samples

Fresh fecal samples (0.1 g) from hamsters were inactivated by 0.2 ml MeOH. After homogenization, 3 aliquots (250 µL in each vial) were taken for bile acid, tryptophan metabolites and SCFA analysis, respectively. Extraction steps were carried out at 4°C to avoid the degradation of compounds, especially for SCFAs. Samples were extracted and analyzed as follows. *Tryptophan metabolites*: 50 µl internal standard in MeOH and 500 µl H_2_O were added to the 450 µl fecal extracts. After 20 minutes agitation at 4°C the samples were centrifugated at 12,000 *g* and 450 µl MeOH were added to the pellet for homogenization and centrifugation. The two supernatants were pooled and evaporated under nitrogen. The dried residues were dissolved in 600 µl MeOH and 3 µl were injected. The LC-MS/MS procedure was performed as previously described.^61^ Mass spectra were obtained using an 5500 Q-Trap (Sciex, Concord, Ontario, Canada) equipped with a TurboIon electrospray (ESI) interface set in the negative mode (needle voltage +5000 V) with nitrogen as the nebulizer set at 40 (arbitrary pressure unit given by the equipment provider). Curtain and heater pressures were set at 20 and 40, respectively (arbitrary unit). The ion source temperature was set at 350°C. Declustering and entrance potentials were set at +60 V and +10 V, respectively. The MS/MS detection was operated at unit/unit resolution. The acquisition dwell time for each transition monitored was 25 ms. Data were acquired by the Analyst® software (version 1.6.2, -Sciex) in the Multiple Reaction Monitoring (MRM) mode. *Bile acid molecular species*. 2 ml of 0.2 M NaOH were added to the 450 µl fecal methanol extracts. After 20 minutes agitation at 60°C, 4 ml H_2_O containing internal standard were added. The whole samples were centrifuged at 12,000 *g* and the supernatants were collected. Final extraction and Mass spectrometry analysis were performed as already described.^62^ *SCFAs*: sample preparation was adapted from protocol of Zheng and collaborators.^63^ Approximatively 30 mg of fecal samples were used and suspended with 1050 µl of a solution of NaOH at 0.005 M including internal standard mix of acetate-D3, butyrate-13C2 and valerate-D9 at 61 µM and ceramic beads. Samples were homogenized at 6500 rpm, 3 × 20s using Prescellys® Evolution (Bertin Technologies, Montigny-le-Bretonneux, France). 300 µl of each supernatant were collected and transferred to 5 ml glass tube. 500 µl of propanol/pyridine mix (3:2 v/v) were added and then vortexed. 50 µl of PCF was successively added twice to the solution and vortexed. Mixtures were sonicated and centrifuged at 2000 x g and 4°C during 5 min. 200 µl of organic phase were transferred to GC/MS vials before their injections. SCFAs in fecal samples were quantified by Gas Chromatographic/mass spectrometry using an ISQ LT™ equipped with a Triplus RSH (Thermo Fisher Scientific, Illkirch, France). A fused-silica capillary column with a (5%-phenyl)-methylpolysiloxane phase (DB-5 ms, J&W Scientific, Agilent Technologies Inc., USA) of 50 m x 0.25 mm i.d coated with 0.25 µm film thickness was used. Temperatures of the front inlet, MS transfer line, and electron impact ion source were set at 260°C, 290°C, and 230°C, respectively. Helium was supplied with carrier gas at a flow rate of 1 ml/min. Oven temperature was set initially at 50°C during 1.5 min. Temperature was raised to 70°C at 8°C/min and to 85°C at 6°C/min. Then, temperature was successively elevated to 110°C at 22°C/min and to 120°C at 12°C/min. Oven temperature was finally set to 300°C at 125°C/min and held 3 min. The run time was 15 min in targeted SIM mode. Injected sample volume was set to 1 µl in split mode with a 20:1 ratio. Data processing was performed using Xcalibur® software (version 3.0, Thermofisher Scientific, Illkirch, France)

### Statistical analyses

Results are expressed as the mean ± standard deviation (SD) unless otherwise stated. All statistical analyses were performed using GraphPad Prism v6 software. A Mann-Whitney *U* test was used to compare two groups unless otherwise stated. Comparisons of more than two groups with each other were analyzed with the Two or One-way ANOVA Kruskal-Wallis test (nonparametric), followed by the Dunn’s posttest.

## Supplementary Material

Supplemental Material

## Data Availability

DOI: 10.5281/zenodo.6342677 (https://zenodo.org/record/6342677)

## Abbreviations

COVID-19, coronavirus disease 2019; SARS-CoV-2, severe acute respiratory syndrome coronavirus 2; NASH, nonalcoholic steatohepatitis; SCFAs, short-chain fatty acids; BA, bile acids RT-PCR, reverse transcription polymerase chain reaction; IL, interleukin; MaAsLin2, microbiome multivariable association with linear models; TCID_50_, tissue culture infectious dose 50.
