# Supplementary figures and images for: Alteration of the gut microbiota’s composition and metabolic output correlates with COVID-19-like severity in obese NASH hamsters
Source: Gut Microbes. 2022 Jul 13;14(1):2100200. doi: 10.1080/19490976.2022.2100200 (PMC9291689; doi:10.1080/19490976.2022.2100200)

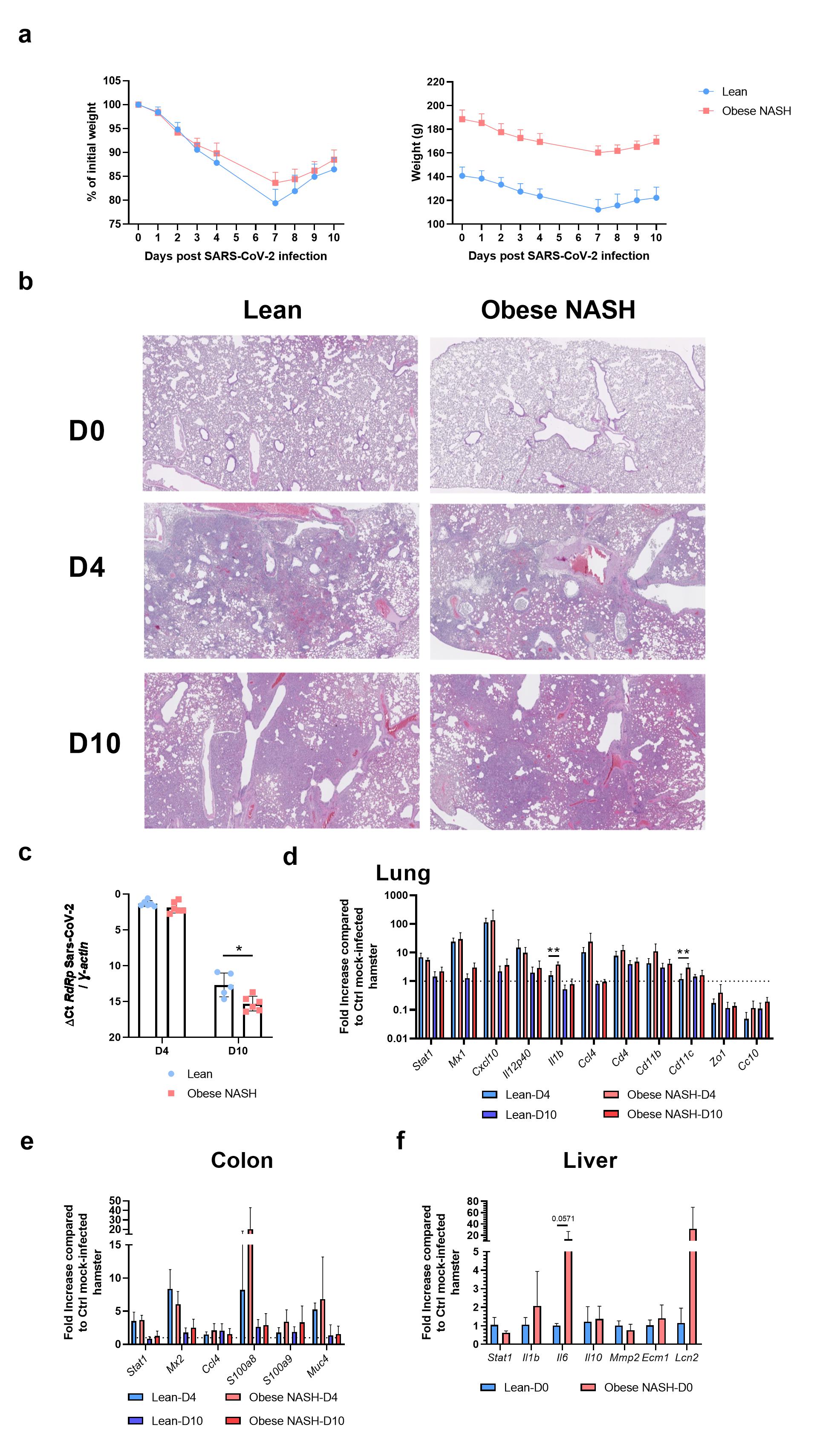

Supplement: Supplemental Material [file KGMI_A_2100200_SM9573.zip › Figure S1.jpg]

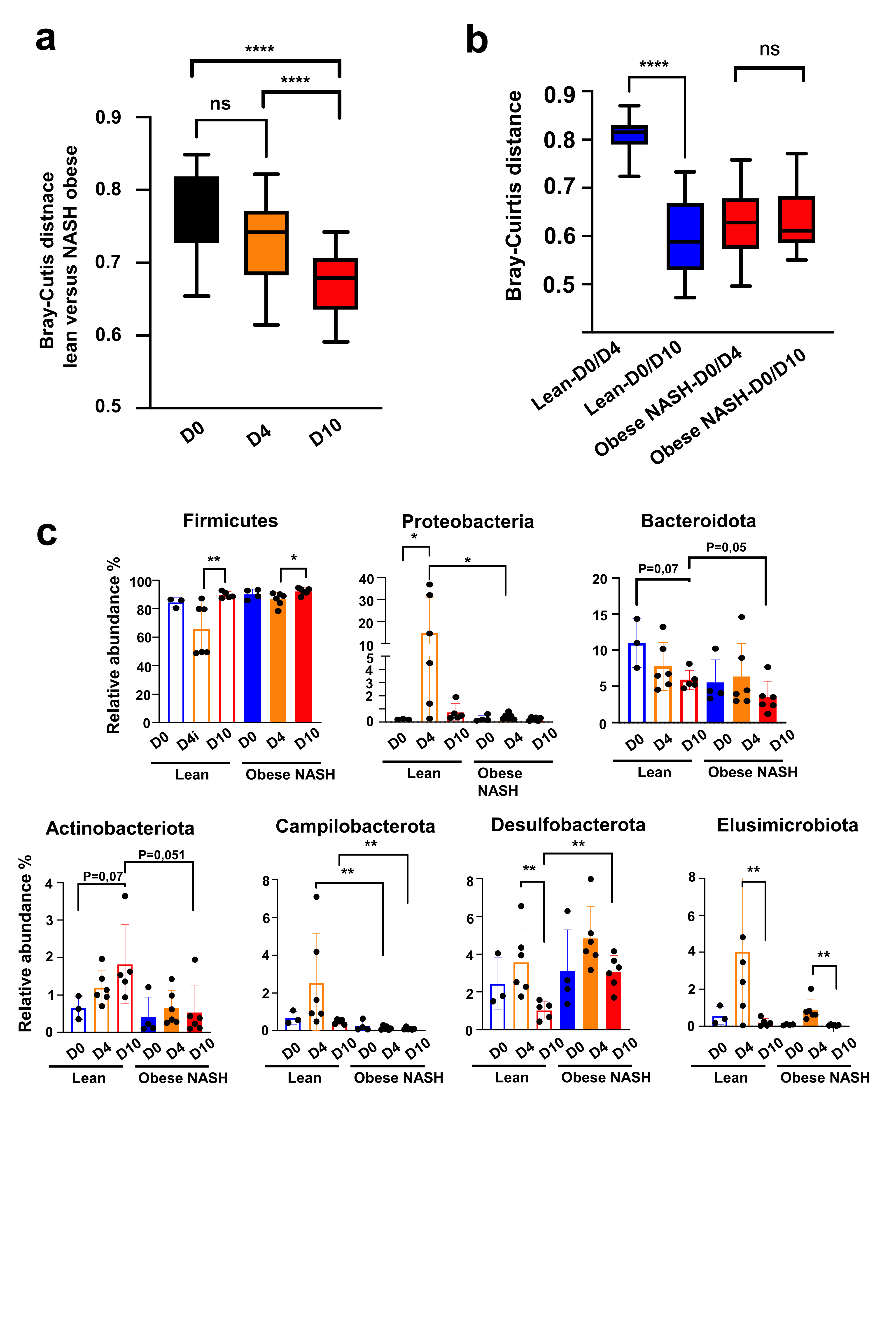

Supplement: Supplemental Material [file KGMI_A_2100200_SM9573.zip › Figure S2 (2).tif]

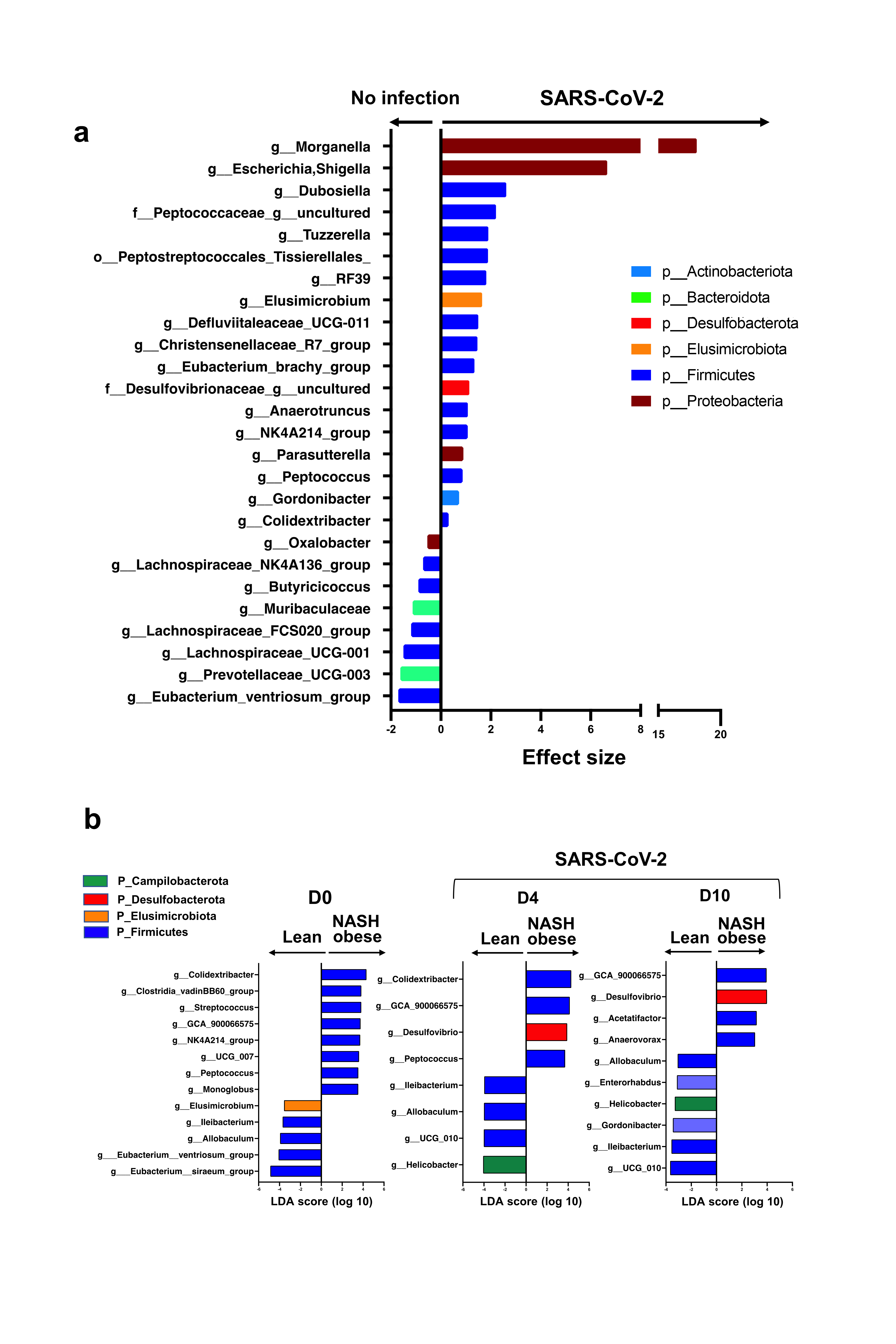

Supplement: Supplemental Material [file KGMI_A_2100200_SM9573.zip › Figure S3 (2).tif]

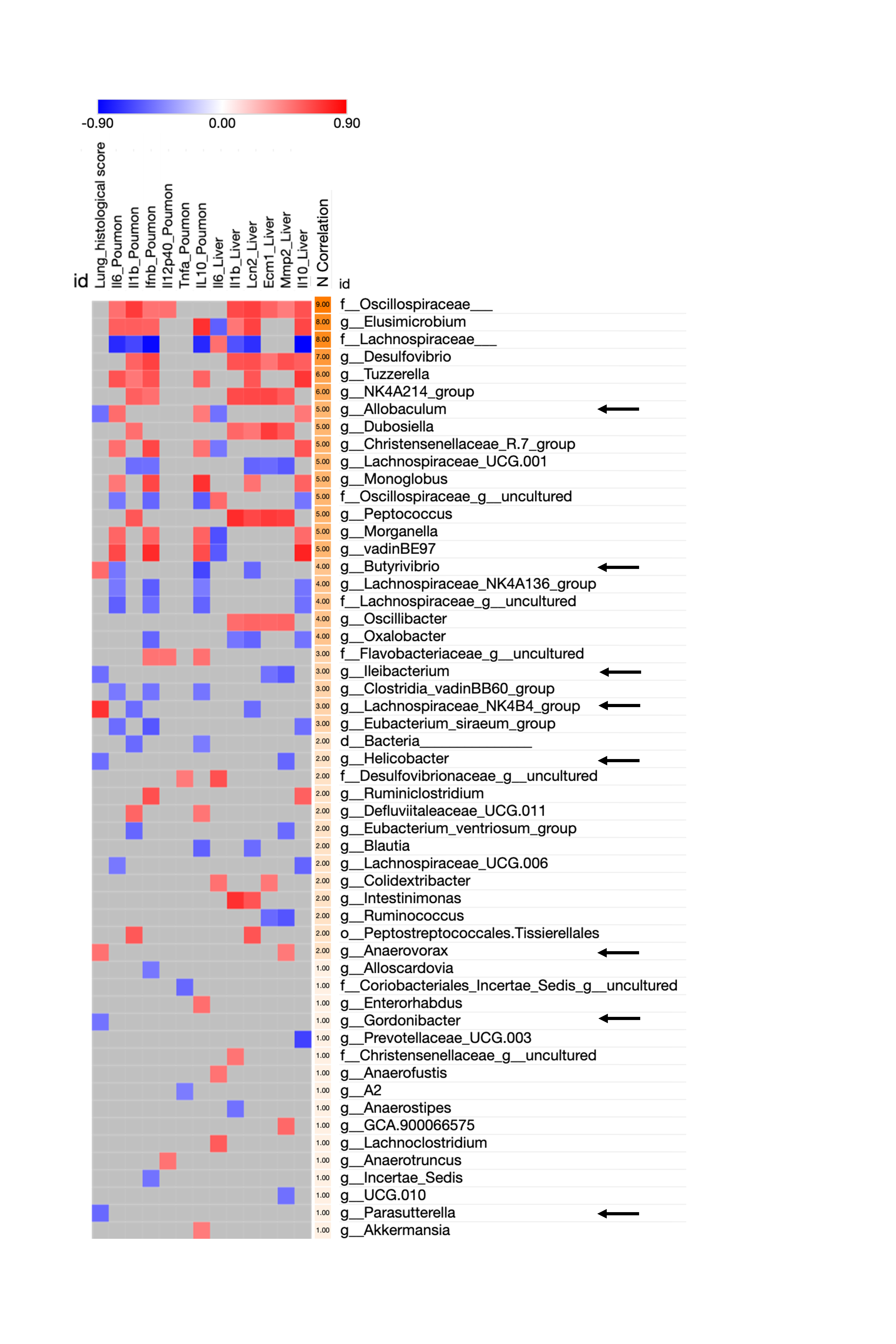

Supplement: Supplemental Material [file KGMI_A_2100200_SM9573.zip › Figure S4 (2).tif]
